# Performance Assessment of Sysmex DI-60: Is Digital Morphology Analyzer Reliable for White Blood Cell Differentials in Body Fluids?

**DOI:** 10.3390/diagnostics14060592

**Published:** 2024-03-11

**Authors:** Eunju Shin, Mina Hur, Hanah Kim, Gun-Hyuk Lee, Mi-Hyun Hong, Minjeong Nam, Seungho Lee

**Affiliations:** 1 Department of Laboratory Medicine, Konkuk University School of Medicine, Seoul 05030, Republic of Korea; shinej0901@naver.com (E.S.); md.hkim@gmail.com (H.K.); leegunhyuk93@gmail.com (G.-H.L.);; 2 Department of Laboratory Medicine, Korea University Anam Hospital, Seoul 02841, Republic of Korea; mjnam0906@gmail.com; 3 Department of Preventive Medicine, Dong-A University College of Medicine, Busan 49315, Republic of Korea; lgydr1@gmail.com

**Keywords:** digital morphology analyzer, DI-60, body fluid, white blood cell differential, performance, turnaround time

## Abstract

Background: Few studies have evaluated digital morphology (DM) analyzers on body fluids (BF). We evaluated the performance of a DM analyzer, Sysmex DI-60 (Sysmex, Kobe, Japan) for white blood cell (WBC) differentials in BF samples. Methods: In five BF samples (two pleural fluids and three ascites) containing a single, dominant cell type (>80%, neutrophils, lymphocytes, macrophages, abnormal lymphocytes, and malignant cells in each sample), we evaluated the precision of the DI-60 and compared the WBC differentials and turnaround times (TAT) between DI-60 and manual counting. Results: The precision of the DI-60 pre-classification and verification was excellent (%CV, 0.01–3.16%). After verification, the DI-60 showed high sensitivity, specificity, and efficiency (ranges: 90.8–98.1%, 96.8–97.9%, and 92.5–98.0%, respectively) for the dominant cell types in neutrophil- and lymphocyte-dominant samples. For all samples, the DI-60 and manual counting showed high correlations for major cell types (neutrophils, lymphocytes, macrophages, and others, r = 0.72 to 0.94) after verification. The agreement between the pre-classification and verification of the DI-60 was strong in the neutrophil-dominant sample (κ = 0.81). The DI-60 showed a significantly longer TAT (min: s) than manual counting for all samples (median TAT/slide: 6:28 vs. 1:53, *p* < 0.0001), with remarkable differences in abnormal lymphocyte- and malignant cell-dominant samples (21:05 vs. 2:06; 12:34 vs. 2:25). Conclusions: The DI-60 may provide reliable data in neutrophil- and lymphocyte-dominant BF samples. However, it may require longer times and higher workloads for WBC differentials especially in BF samples containing atypical cells. Further improvement would be needed before applying DM analyzers for routine clinical practice in BF analysis.

## 1. Introduction

The application of digital morphology (DM) analyzers has been increasing in the clinical practice for the peripheral blood smear (PBS) examination [1,2,3,4]. Numerous studies have reported that DM analyzers may show reliable performances for white blood cell (WBC) counts and differentials after verification [5,6,7,8,9,10,11,12,13,14]. DM analyzers are also expected to reduce the workloads and turnaround times (TAT) of PBS examinations in clinical laboratories [5,9,10,11,13].

In body fluid (BF) analyses, the WBC count and differential are also essential, and increased WBC counts as well as dominant cell types could help physicians to differentiate diseases, such as viral or bacterial infections, inflammation, metastasis, and lymphoproliferative disorders [15]. Although manual counting has been the gold standard for WBC counting and differentials, not only in PBs, but also in BF, it is time consuming, labor intensive, and requires the examiners’ proficiency [16,17,18,19]. To reduce the workload and TAT in laboratories, numerous studies have evaluated the performances and feasibilities of automated hematology analyzers on BF [15,16,17,20,21,22]. While DM analyzers can be used for WBC counts and differentials in BF samples, only a few such studies have been reported so far [18,19,23,24]. Riedl et al. [18] first analyzed BFs using a CellaVision DM96 (CellaVision AB, Lund, Sweden) and showed a high pre-classification accuracy and a high correlation for post-classification (verification) and manual counting.

The Sysmex DI-60 (DI-60; Sysmex, Kobe, Japan) is one of the most commonly used DM analyzers in clinical laboratories [3]. The DI-60 can pre-classify cells into a total of 8 classes in BF mode (neutrophils, lymphocytes, eosinophils, macrophages, others, unidentified, smudge cells, and artifact), and it pre-classifies cells into a total of 18 classes in PB mode [25]. Although there have been only three studies that have evaluated the performance of the DI-60 on BF, these previous studies were confined to partial cell classes (one to five classes) [19,23,24]. To our knowledge, no study has evaluated the performance of the DI-60 for all eight classes or compared DI-60 pre-classification, verification, and manual counting. Moreover, the TAT of the DI-60 in BF analysis has never been evaluated. In this study, we comprehensively evaluated the performance of DI-60 pre-classification and verification in comparison with manual counting. We also compared the TAT between the DI-60 and manual counting on BF samples. Given the fewer cell classes in the BF mode than in the PB mode, we hypothesized that DI-60 pre-classification would be more efficient and straightforward on BF than that on PB, and the TAT of DI-60 verification would be shorter than that of manual counting.

## 2. Materials and Methods

### 2.1. Study Samples

This in vitro evaluation study was conducted from January to March 2023 at the Konkuk University Medical Center (KUMC), Seoul, Korea. This study was performed using remnant samples after a requested BF analysis and required neither study-specific intervention nor additional BF collection. Therefore, the Institutional Review Board of KUMC approved the study protocol (KUMC 2023-05-021) and waived informed consent.

**Figure 1:**
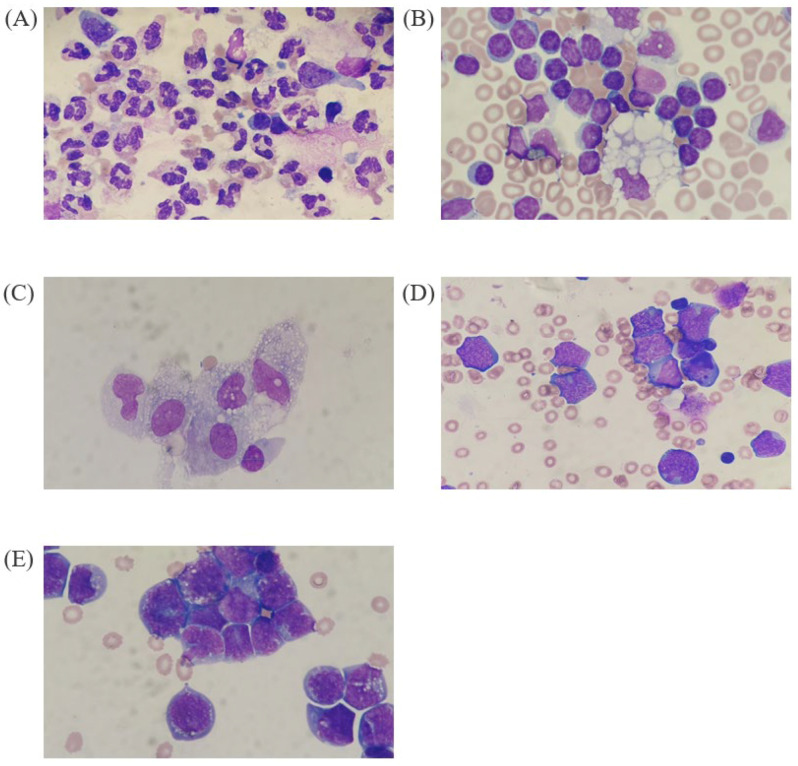
Five body fluid samples containing a single, dominant cell type (under a high power of 1000×). (**A**) Neutrophil-dominant (85 yr old female, pneumonia, pleural fluid), (**B**) lymphocyte-dominant (96 yr old male, pneumonia, pleural fluid), (**C**) macrophage-dominant (63 yr old male, liver cirrhosis and hepatocellular carcinoma without metastasis, ascites), (**D**) abnormal lymphocyte-dominant (21 yr old male, burkitt lymphoma, ascites), (**E**) malignant cell-dominant (69 yr old female, ovary small carcinoma, ascites).

We used five BF samples (two pleural fluids and three ascites) that contained a single, dominant cell type (>80%, neutrophils, lymphocytes, macrophages, abnormal lymphocytes, and malignant cells in each sample). We selected these samples intentionally, because we wanted to explore the performance of the DI-60, under the condition that it can guarantee easy and intuitive cell distinction if performed by experienced hematology experts. Figure 1 shows the five BF slides that contained a single, dominant cell type along with the clinical information of each patient. All BF samples were run using the BF mode on a Sysmex XN-9000 (XN-BF; Sysmex, Kobe, Japan). The XN-BF differentiates the WBC in BF samples into four cell types (neutrophils, lymphocytes, eosinophils, and monocytes) and indicates both the percentage (%) and absolute values [15]. The five BF slides were prepared using a Cytospin 4 centrifuge (Thermo Fisher Scientific, Waltham, MA, USA); after cytocentrifugation for 5 min at 1500 rpm, the slides were stained with Wright–Giemsa using an SP-50 (Sysmex).

### 2.2. WBC Differential in BF Using DI-60 and Manual Counting

A DI-60 consists of a microscope, digital camera, and a computer system with CellaVision Remote Review software (version 7.0.1) that can acquire pre-classified cell images from a slide [4,5,7]. The BF analysis of DI-60 is designed to utilize cytocentrifuge-prepared slides and digitally scan the entire smear area at 10× or at 50× magnification [25]. The DI-60 pre-classifies a total of eight classes (six WBC classes and two non-WBC classes) in an automatically selected area. The six WBC classes include neutrophils, lymphocytes, eosinophils, macrophages (including monocytes), others (basophils, lymphoma cells, atypical lymphocytes, blasts, and tumor cells), and unidentified cells; the two non-WBC classes include smudge cells and artifacts [25]. In an overview application, examiners can select the area of interest manually and detect abnormal cells more easily [23].

Using the DI-60, a WBC differential was conducted 10 times consecutively on each of the five BF slides. The results pre-classified by DI-60 were verified by an experienced hematology expert. The DI-60 completed analysis, when 200 cells were counted during the pre-classification. Non-WBCs (smudge cells and artifacts) and unidentified cells were not included in the setting of the 200-cell count; accordingly, actual cell counts might be more than 200 cells/run, including non-WBCs and unidentified cells.

The manual counting for the WBC differential was performed according to the CLSI H56-A guidelines [26]. One experienced hematology expert scanned slides using a low-power (100×) microscope and counted 200 WBCs after switching to high-power magnification (400×). Non-WBCs and unidentified cells were additionally counted for the evaluation in the same condition, while the 200 WBCs were counted. The 200 WBCs were classified into five WBC classes (neutrophils, lymphocytes, eosinophils, macrophages, and others).

### 2.3. Assessment of TAT

The TAT of the DI-60 was evaluated using the log data of the DI-60 that included the time of preparing for scanning, scanning the ideal zone, pre-classification, and verification. The TAT of manual counting was evaluated manually, including the time of placing a slide on the microscope, scanning the ideal zone, counting cells, and recording results. The total TAT value was the sum of the median TATs for the cell counting per slide in each step.

### 2.4. Statistical Analysis

The number of cells from the DI-60, XN-BF, and manual counting were expressed as a median (interquartile range, IQR) or number (percentage, %). For precision, each slide was run 10 times using the DI-60 and by manually counting [27]. Repeatability was expressed as the standard deviation (SD) and % coefficients of variation (%CVs). %CVs were interpreted as follows: %CV ≤ 10%, excellent; %CV 10–20%, good; %CV 20–30%, acceptable; %CV > 30%, poor [28].

The performances of the DI-60 pre-classification and verification were evaluated using the value of manually counting as a gold standard (true value). The sensitivity, specificity, and efficiency were calculated according to the CLSI EP12-Ed3 guidelines [29]. A Bland–Altman plot and Passing–Bablok regression analysis were used for comparisons between the DI-60 and manual counting, according to the CLSI EP09c-Ed3 guidelines [30]. Using the Bland–Altman plot, the absolute mean difference (%) was interpreted informally to visualize the difference between the DI-60 (both pre-classification and verification) and manual counting. The four major cell classes (neutrophils, lymphocytes, macrophages, and others) between the DI-60 and manual counting were compared using Passing–Bablok regression. In the regression equation, the slope and intercept were calculated with their 95% confidence intervals (CIs), and these CIs were used to determine whether there is a chance difference between the slope and 1 and between the intercept and 0. Pearson’s correlation coefficients (r) with a 95% CI were interpreted as follows: <0.30, negligible; 0.30–0.50, low; 0.50–0.70, moderate; 0.70–0.90, high; 0.90–1.00, very high [31]. The agreement between the DI-60 pre-classification and verification was calculated using Cohen’s kappa (κ) with a 95% CI, which was interpreted as follows: ≤0.20, none; 0.21–0.39, minimal; 0.40–0.59, weak; 0.60–0.79, moderate; 0.80–0.90, strong; and >0.90, nearly perfect [32]. The total TATs per slide between the DI-60 and manual counting were analyzed using an independent sample *t*-test.

Statistical analyses were performed using Microsoft Excel Software (version 2016; Microsoft Corporation, Redmond, WA, USA) and MedCalc Statistical Software (version 22.001; MedCalc Software, Ostend, Belgium). *p* values < 0.05 were considered statistically significant.

## 3. Results

The DI-60 counted 243.5 cells (IQR, 217–451.8 cells) per slide. The repeatabilities of the DI-60 pre-classification and verification were excellent in all eight cell classes (%CV, 0.01–3.16%). Macrophages in the macrophage-dominant sample showed the lowest %CV (0.01%), and eosinophils in the neutrophil- and macrophage-dominant samples showed the highest %CVs (3.16%). The absolute mean difference ranged from 0% to 56.38% in pre-classification vs. manual counting, and from 0% to 18.99% in verification vs. manual counting. In all samples, the absolute mean difference between the DI-60 and manual counting decreased after verification (pre-classification vs. verification; 5.87% vs. 4.68% in neutrophil-dominant sample; 40.09% vs. 18.99% in lymphocyte-dominant sample; 56.38% vs. 17.18% in abnormal lymphocyte-dominant sample; 53.24% vs. 11.35% in malignant-cell dominant sample), except for in the macrophage-dominant sample (3.38% vs. 3.79%) (Table 1).

After verification, the DI-60 showed high sensitivity, specificity, and efficiency (range, 90.8–98.1%, 96.8–97.9%, and 92.5–98.0%, respectively) for dominant cell types in neutrophil- and lymphocyte-dominant samples. For dominant cell types in macrophage-, abnormal lymphocyte-, and malignant cell-dominant samples, the DI-60 showed increased sensitivity (from 99.3% to 100%, from 38.4% to 100%, and from 24.7% to 100%, respectively) and decreased specificity (from 92.6% to 74.9%, from 100% to 68.6%, and from 100% to 78.1%, respectively) after verification. The DI-60 showed decreased efficiency for dominant cell types in macrophage- and abnormal lymphocyte-dominant samples (from 98.0% to 95.2% and from 84.4% to 76.6%, respectively) after verification, while it showed increased efficiency for the dominant cell type in the malignant cell-dominant sample (from 71.8% to 86.3%) (Table 2).

**Figure 2:**
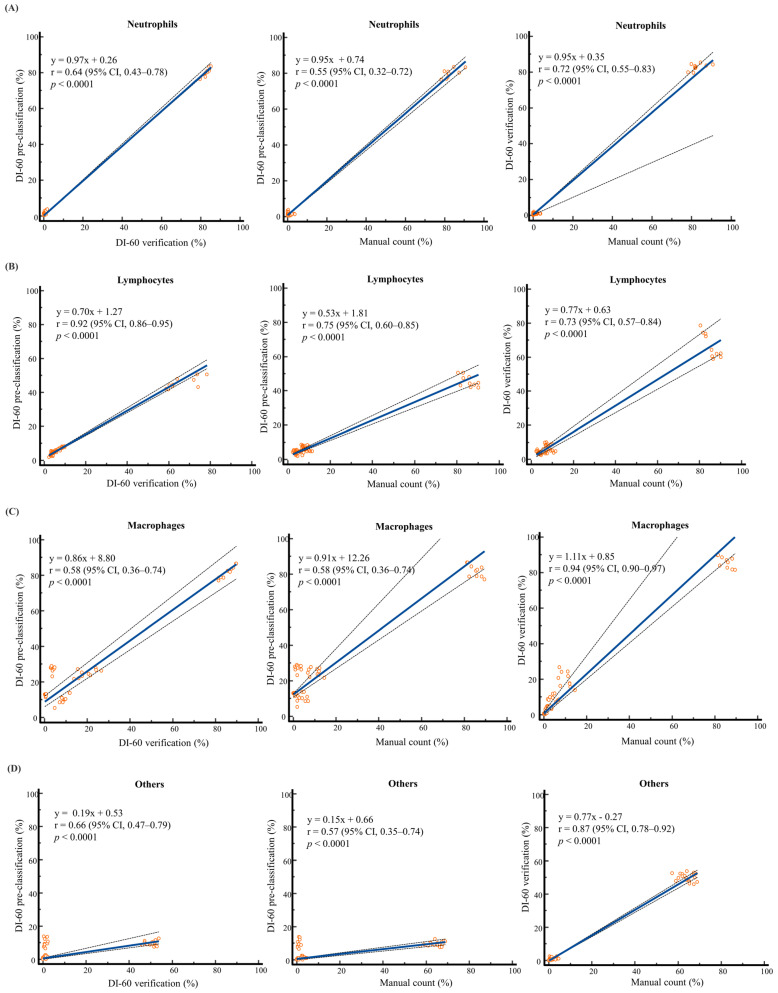
Comparison of major cell types between DI-60 and manual counting (n = 50): (**A**) neutrophils, (**B**) lymphocytes, (**C**) macrophages, and (**D**) others. WBC differentials on five body fluid samples were conducted 10 consecutive times on each slide using the DI-60 and by manually counting. Others include basophils, lymphoma cells, atypical lymphocytes, blasts, and tumor cells. Solid line, Passing–Bablok regression; dashed line, 95% CI line. Abbreviations: n, number; WBC, white blood cell; CI, confidence interval.

The pre-classification of the DI-60 and manual counting showed a high correlation for lymphocytes (r = 0.75) and moderate correlations for neutrophils (r = 0.55), macrophages (r = 0.58), and others (r = 0.57). After verification, the DI-60 and manual counting showed high to very high correlations for neutrophils (r = 0.72), lymphocytes (r = 0.73), macrophages (r = 0.94), and others (r = 0.87). The pre-classification and verification showed very high correlations for lymphocytes (r = 0.92) and moderate correlations for neutrophils (r = 0.64), macrophages (r = 0.58), and others (r = 0.66) (Figure 2).

The agreement between the pre-classification and verification of the DI-60 was strong in the neutrophil-dominant sample (κ = 0.81) and minimal in the abnormal lymphocyte- and malignant cell-dominant samples (κ = 0.32 and 0.29, respectively). In the abnormal lymphocyte- and malignant cell-dominant samples, the DI-60 misclassified ‘others’ (82.0% and 86.8%, respectively) into various cell types (Table 3).

**Figure 3:**
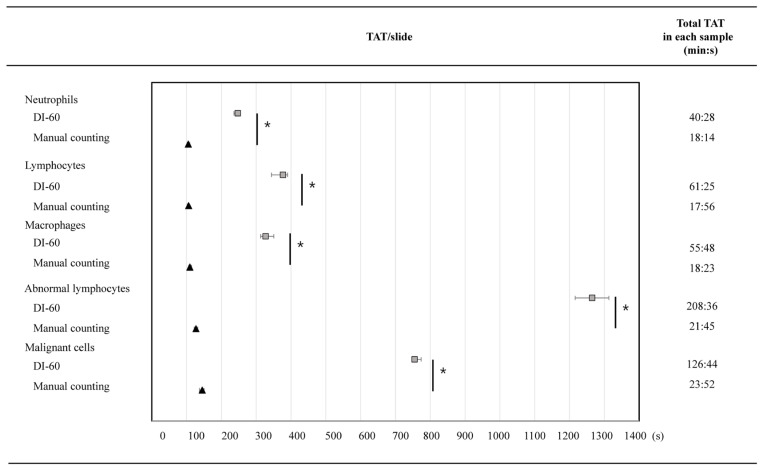
Comparison of TAT/slide between the DI-60 and manual counting for the five body fluid samples. Squares indicate the median TAT of the DI-60, triangles indicate the median TAT of the manual counting, and lines indicate interquartile ranges. Total TAT means the sum of 10 TATs/slide in each group. * *p* < 0.0001. Abbreviation: TAT, turnaround time.

The TAT of the DI-60 was significantly longer than that of manual counting in all five BF samples (*p* < 0.0001). In the DI-60, the median TAT/slide and total TAT were over nine folds longer than those of manual counting (1265 vs. 126 s; 208:36 vs. 21:45 min: s) in the abnormal lymphocyte-dominant sample and about five folds longer than those of manual counting in the malignant cell-dominant sample (754 vs. 145 s; 126:44 vs. 23:52 min: s) (Figure 3). Due to the TATs of the pre-classification and verification, the abnormal lymphocyte- and malignant cell-dominant samples showed remarkable differences in TAT between the DI-60 and manual counting (min: s, 21:05 vs. 2:06; 12:34 vs. 2:25, *p* < 0.0001) (Table 4).

## 4. Discussion

In this study, we comprehensively evaluated the performance of a DI-60 for WBC differentials in all eight classes of BF mode and compared the DI-60 (pre-classification and verification) with manual counting, including its TAT. In real-world practice, most BF samples are composed of heterogeneous cell types including tissue cells; these cells may affect the accuracy of a WBC differential in automated hematology analyzers [20]. Therefore, we intentionally used five BF samples that contained a single, dominant cell type (over 80% in each sample). Given that WBC differentials in samples with rather homogenous dominant cell types could be performed straightforwardly by experienced hematologists, using such samples would be the fundamental step for the distinction of cell morphology in DM analyzers.

In our data, the precisions of the DI-60 pre-classification and verification were excellent (%CV, 0.01–3.16%), and the %CV of dominant cells showed higher precisions, especially in neutrophils-, lymphocytes-, and macrophages-dominant samples (Table 1). The DI-60 also showed high sensitivity, specificity, and efficiency after verification for dominant cell types in neutrophil- and lymphocyte-dominant samples (Table 2). However, in abnormal lymphocyte- and malignant cell-dominant samples, the ‘others’ class showed the biggest absolute mean difference between DI-60 pre-classification and manual counting and low sensitivities in DI-60 pre-classification (Table 1 and Table 2).

To our knowledge, there have been only three studies that have evaluated a DI-60 on the WBC differentials in BF [19,23,24]. Differently from previous studies that were confined to partial cell class evaluations, we evaluated the performance of the DI-60 in BF for all eight classes. Moreover, none of the previous studies have compared the TAT between a DI-60 and manual counting. Takemura et al. [19] first evaluated DI-60 post-classification compared with manual counting for only four cell types (neutrophils, lymphocytes, eosinophils, and monocytes). They showed a good correlation between the two methods except for monocytes. In our study, the correlation between DI-60 verification and manual counting was high to very high for neutrophils, lymphocytes, macrophages, and even others (Figure 2). Yamatani et al. [23] additionally evaluated the performance of a DI-60 for only tumor cell detection. They compared three methods (manual counting, DI-60 post-classification, and the DI-60 overview application) and showed that the DI-60 overview application had higher sensitivity and specificity than manual counting and DI-60 post-classification. Although we did not use the DI-60 overview application, our study also showed the high sensitivity of the DI-60 verification in another class of the malignant cell-dominant sample.

Yoon et al. [24] recently evaluated the analytical performance of the DI-60 for five cell types (neutrophils, lymphocytes, eosinophils, macrophages, and ‘other’ cells) and compared the DI-60 with manual counting for four cell types (neutrophils, lymphocytes, eosinophils, and macrophages). They reported that the DI-60 misclassified ‘other’ cells into various cell types (66.3%), especially into macrophages [24]. In our study, the abnormal lymphocyte- and malignant cell-dominant samples were mainly composed of ‘other’ cells, and the DI-60 misclassified most of the “others” (78.3% and 86.5%, respectively) into macrophages, artifacts, or unidentified (Table 3). Due to the fewer cell classes in BF mode, the ‘others’ class in BF may contain more various and heterogenous abnormal cells at a time, leaving further sophisticated classification to be conducted at the verification stage [25]. Therefore, we can assume that DI-60 pre-classification on BF can be performed more straightforwardly than that on PB. However, compared with a previous study finding [7], our study showed that the agreement in BF samples containing many ‘others’ was lower (κ = 0.32 and 0.29) than the agreement for immature granulocytes and blasts (κ = 0.43 and 0.54, respectively) in PB. These findings might be attributed to many smudge cells and artifacts in the abnormal lymphocyte- and malignant cell-dominant samples that could increase the chance of misclassification in DM analyzers. Furthermore, the more ‘other’ cells, smudge cells, and artifacts that exist in BF samples, the longer the time needed for the pre-classification and verification. Our data underscore that DI-60 verification is an essential process for re-classifying misclassified cells; it is also noteworthy that the verification process could increase laboratory workloads.

The manufacturer-claimed throughput of the DI-60 in BF analysis is up to 40 cytospin slides/hour (100 WBCs at 10×) and 5 cytospin slides/hour (100 WBCs at 10× and 50×) [25]. In our study, 200 WBCs were counted by the DI-60, and the manufacturer’s claimed throughput was met for all five samples. However, the throughput would be highly dependent on sample composition; therefore, TAT is one of the essential parts for assessing laboratory efficiency [9]. In our study, the DI-60 showed significantly longer TATs than manual counting for all samples, especially for abnormal lymphocyte- and malignant cell-dominant samples. The DI-60 also needed a longer time in all four steps compared with manual counting (Table 4 and Figure 3). In general, the purpose of using a DM analyzer is not only to increase accuracy, but also to decrease workload. However, our study demonstrated that the additional verification process prolonged the TAT. Similarly, several studies on the performances of DM analyzers on PB samples showed longer TATs than that of manual counting [9,10,11]. Our data imply that current DM analyzers may not increase laboratory efficiency as much as expected until the TAT would be shortened. In the meanwhile, considering that there are fewer and fewer specialists in laboratories who can assess cells in BF, both those with normal morphology and pathological cell types, despite the longer TAT completion time, the DI 60 may be a great help in routine diagnostics.

In this study, our analysis was based not on the five slides but on the 50 replicated measurements, and a total of 30,408 cells were actually counted for both DI-60 verification and manual counting; it was a sufficient number of measurements for exploring the analytical performance of the DI-60 using BF. This study also has several limitations. First, we focused on WBC differentials only, although DM analyzers provide information on RBCs as well [15]. Second, we used pleural fluids and ascites except cerebrospinal fluid (CSF), because we needed a sufficient cell count for the dominant cell type. CSF samples usually show extremely low cell counts that increase the imprecision of a DM analyzer and manual counting [20]. Third, we evaluated only the TAT of the DI-60, although risk assessment is another key indicator of laboratory efficiency [33]. Nam et al. [9] showed that the DI-60 has a five-fold lower risk than manual counting on leukopenic PB samples. Recently, there has been emerging DM analysis, even without an expert’s review [34]. However, the results of laboratory tests should be considered comprehensively, including the type of sample, sample quality, and disease of the patient. Moreover, standardization and guidelines for DM analysis have not been established [35]. Taken together, further research and improvements are anticipated for the complete implementation of DM analyzers in clinical laboratories.

## 5. Conclusions

In conclusion, this is the first study that comprehensively evaluated the performance of a DI-60 for WBC differentials in BF samples. WBC differentials with the DI-60 on BF seem to be reliable and promising but not yet perfect, especially with challenging samples. Considering that this evaluation was conducted using BF samples with a single, dominant cell type, the data in real-world practice would show more remarkable differences and larger gaps, using various BF samples with heterogenous cell compositions. The prolonged TAT of the DI-60 compared with that of manual counting may leave a question about whether current DM analyzers would indeed streamline laboratories’ workflows and improve laboratory efficiency. Applying DM analyzers for routine practice in BF analysis would be feasible with further improvement.

## Figures and Tables

**Table 1:** WBC differentials from DI-60 and manual counting with DI-60 precision.

BF Samples	Cell Class	XN-9000	DI-60		Manual Count	DI-60 Repeatability		Mean Difference (%, 95% CI)	
		Cell n/μL (%)	Cell n/Slide, Median (IQR)		Cell n/Slide, Median (IQR)	SD (%CV)		Pre-Classification vs. Manual Counting	Verification vs. Manual Counting
			Pre-Classification	Verification		Pre-Classification	Verification		
Neutrophil-dominant	N	672 (86.9)	168 (162.8–170.8)	173.5 (171.3–176.8)	175 (174–180.5)	5.21 (0.03)	4.11 (0.02)	−3.13 (−8.51 to 2.26)	−0.27 (−6.30 to 5.77)
	L	36 (4.7)	7.5 (5.3–9.8)	9 (7.5–9.8)	8 (6.5–8.8)	2.67 (0.35)	2.07 (0.24)	−0.28 (−4.78 to 4.22)	0.20 (−3.50 to 3.89)
	E	1 (0.1)	1 (0–1)	0 (0–0)	0 (0–0)	0.67 (0.96)	0.63 (3.16)	0.29 (−0.37 to 0.94)	0.10 (−0.49 to 0.68)
	M	64 (8.3) ^†^	21.5 (18–22.8)	18 (17–20.8)	9 (5.3–11.8)	5.38 (0.26)	4.50 (0.24)	5.87 (0.54 to 11.21)	4.68 (−0.28 to 9.64)
	O *	NA	3 (2–3.8)	2 (1.25–3)	6 (5.3–7.8)	1.52 (0.53)	1.25 (0.54)	−1.57 (−4.09 to 0.95)	−1.86 (−3.86 to 0.15)
	S	NA	5 (3.25–6)	4 (3–4.8)	11.5 (7.8–12)	1.64 (0.35)	1.89 (0.44)	−2.63 (−6.45 to 1.19)	−2.63 (−6.45 to 1.19)
	A	NA	3 (1.25–3)	2 (1.3–3)	2.5 (2–4)	1.35 (0.52)	1.45 (0.69)	−0.07 (−2.16 to 2.02)	−0.32 (−2.17 to 1.55)
	U	NA	3 (2–3)	0 (0–0)	0 (0–0)	0.95 (0.35)	0.42 (2.11)	1.28 (0.41 to 2.16)	0.10 (−0.30 to 0.49)
Lymphocyte-dominant	N	8 (1.2)	2 (2–2)	2 (2–2)	0.5 (0–1)	0.82 (0.41)	0.79 (0.44)	0.46 (−0.53 to 1.45)	0.38 (−0.59 to 1.36)
	L	623 (89.9)	113 (108.5–115.8)	156 (151.8–173.3)	175 (171.3–179.8)	4.98 (0.04)	12.67 (0.08)	−40.09 (−51.92 to −28.25)	−18.99 (−39.34 to 1.36)
	E	0 (0.0)	0.5 (0–1)	0 (0–0)	0 (0–0)	0.70 (1.17)	0.0 (NA)	0.14 (−0.67 to 0.96)	−0.10 (−0.50 to 0.30)
	M	62 (8.9)	62.5 (59–64)	48.5 (41.3–58.8)	23 (16.3–25)	5.80 (0.09)	15.44 (0.30)	14.38 (7.01 to 21.75)	9.35 (−2.75 to 21.46)
	O	NA	24 (21.3–27.8)	2 (1.3–3)	2 (1.3–2.8)	5.28 (0.22)	1.51 (0.60)	8.99 (4.33 to 13.65)	0.15 (−1.60 to 1.90)
	S	NA	6.5 (6–10.5)	5.5 (5–8.8)	3 (3–5.5)	3.02 (0.38)	2.99 (0.46)	1.52 (−0.85 to 3.89)	0.73 (−1.78 to 3.23)
	A	NA	26 (16.5–34.8)	20.5 (12–25.5)	1 (1–2)	14.61 (0.51)	13.47 (0.62)	10.43 (1.77 to 19.09)	8.09 (−1.86 to 18.04)
	U	NA	10 (8.5–11)	1 (0–1)	0 (0–0)	2.82 (0.28)	0.99 (1.10)	4.17 (1.71 to 6.62)	0.37 (−0.46 to 1.20)
Macrophage-dominant	N	14 (14.6)	4.5 (4–6)	1 (1–2.8)	0 (0–1)	1.85 (0.38)	1.35 (0.84)	1.95 (0.24 to 3.66)	0.48 (−0.85 to 1.81)
	L	39 (40.6)	11 (10–12.8)	10.5 (8.3–11.8)	17 (14.5–21.5)	1.48 (0.13)	2.00 (0.19)	−3.20 (−8.14 to 1.74)	−3.57 (−8.22 to 1.07)
	E	0 (0.0)	0 (0–0)	0 (0–0)	0 (0–0)	0.32 (3.16)	0.00 (NA)	0.04 (−0.22 to 0.31)	0.00 (0.00 to 0.00)
	M	43 (44.8)	183.5 (183–184.8)	192 (192–193.8)	181.5 (177.8–185.3)	2.06 (0.01)	2.62 (0.01)	−3.38 (−14.69 to 7.93)	0.58 (−10.41 to 11.56)
	O	NA	0 (0–1)	0 (0–0)	0.5 (0–1)	0.71 (1.41)	0.00 (NA)	−0.05 (−0.97 to 0.87)	−0.28 (−0.94 to 0.37)
	S	NA	6 (5–7)	3 (2–3)	6 (4.3–6.8)	1.66 (0.27)	1.60 (0.55)	0.17 (−1.81 to 2.15)	−1.25 (−3.80 to 1.31)
	A	NA	11.5 (10–23)	11.5 (9.3–25.5)	8 (5.5–9.8)	7.69 (0.49)	9.22 (0.55)	3.37 (−4.01 to 10.74)	3.79 (−4.85 to 12.42)
	U	NA	2.5 (1.25–3)	0 (0–1)	0 (0–0)	1.35 (0.54)	0.84 (1.41)	1.10 (−0.02 to 2.22)	0.26 (−0.45 to 0.96)
Abnormal lymphocyte-dominant	N	17 (10.3)	6 (6–6.8)	4 (4–4.8)	0 (0–1)	0.74 (0.12)	0.63 (0.15)	0.69 (0.26 to 1.12)	0.43 (−0.05 to 0.91)
	L	28 (17.1)	28.5 (27–29.8)	26.5 (25.3–27)	11 (10–11.8)	2.62 (0.09)	2.25 (0.09)	−0.17 (−1.60 to 1.27)	−0.48 (−1.89 to 0.93)
	E	0 (0.0)	1.5 (1–2.8)	0 (0–0)	0 (0–0)	1.34 (0.79)	0.0 (NA)	0.23 (−0.13 to 0.60)	0.00 (0.00 to 0.00)
	M	119 (72.6)	93 (88.5–95.5)	4 (2.5–4.8)	2 (1–2)	4.06 (0.04)	1.69 (0.44)	11.89 (10.42 to 13.36)	−0.11 (−0.90 to 0.67)
	O	NA	70.5 (69.25–4.75)	362.5 (352.3–370.5)	187 (186–187.8)	3.81 (0.05)	19.69 (0.05)	−56.38 (−59.67 to −53.08)	−17.18 (−23.19 to −11.17)
	S	NA	109.5 (103.3–112.5)	195 (188.5–206.3)	67.5 (63–75.5)	6.87 (0.06)	13.94 (0.07)	−9.16 (−15.54 to −2.77)	3.23 (−2.25 to 8.72)
	A	NA	248.4 (230–267.8)	118.5 (123.8–113.5)	7 (6.25–8)	29.84 (0.12)	28.43 (0.23)	30.98 (26.27 to 35.69)	14.51 (7.94 to 21.08)
	U	NA	183 (176.3–188.8)	20 (18.5–23)	8 (6–9)	12.53 (0.07)	4.37 (0.22)	21.91 (18.90 to 24.91)	−0.01 (−1.72 to 1.70)
Malignant cell-dominant	N	109 (4.9)	4.5 (4–6)	4 (4–4)	5 (4.25–6)	1.29 (0.26)	0.57 (0.14)	−1.08 (−2.55 to 0.40)	−1.26 (−2.82 to 0.31)
	L	540 (23.7)	33.5 (31.3–35)	37.5 (35.3–39.5)	20.5 (19–22.5)	3.28 (0.10)	3.99 (0.11)	−0.52 (−3.44 to 2.39)	0.57 (−2.47 to 3.62)
	E	14 (0.6)	0 (0–0)	0 (0–0)	0 (0–0)	0 (NA)	0.0 (NA)	0.0 (0.0 to 0.0)	0.0 (0.0 to 0.0)
	M	1611 (70.8)	121 (120–125.5)	17 (15.5–20)	5 (4.25–7.5)	5.96 (0.05)	2.80 (0.16)	24.99 (21.32 to 28.66)	1.75 (−0.54 to 4.03)
	O	NA	39.5 (37–44)	231 (222.8–234)	169.5 (163.5–170)	7.28 (0.18)	13.18 (0.06)	−53.24 (−60.41 to −46.07)	−11.35 (−18.18 to −4.52)
	S	NA	73.5 (70.3–74.8)	132.5 (125.5–138)	54.5 (48.3–63.5)	4.92 (0.07)	8.79 (0.07)	−3.75 (−10.17 to 2.67)	9.54 (4.32 to 14.76)
	A	NA	85 (81.5–88.3)	18.5 (13–23.3)	7.5 (5.5–10)	6.87 (0.08)	9.30 (0.46)	15.73 (12.78 to 18.69)	0.97 (−3.58 to 5.52)
	U	NA	89 (82.8–92.5)	6 (5–7)	4.5 (4–5)	9.21 (0.11)	2.72 (0.42)	17.87 (14.29 to 21.45)	−0.22 (−1.37 to 0.93)

* Other cells include basophils, lymphoma cells, atypical lymphocytes, blasts, and tumor cells. ^†^ Monocytes counted using a XN-9000 were classified into macrophages. Abbreviations: BF, body fluid; n, number; N, neutrophils; L, lymphocytes; E, eosinophils; M, macrophages; O, other; S, smudge cells; A, artifacts; U, unidentified; NA, not available; IQR, interquartile range; SD, standard deviation; CV, coefficient of variation.

**Table 2:** Performances of DI-60 pre-classification and verification on the basis of manual counting (10 experiment designs for each sample).

BF Samples	Cell Class	Total Cell n		Sensitivity (%)		Specificity (%)		Efficiency (%)	
		Pre-Classification	Verification	Pre-Classification	Verification	Pre-Classification	Verification	Pre-Classification	Verification
Neutrophil-dominant	N	1677	1737	95.1 (94.0–96.0)	98.1 (97.3–98.7)	100 (98.9–100)	97.9 (95.8–99.2)	95.9 (94.9–96.7)	98.0 (97.4–98.6)
	L	76	86	74.7 (64.0–83.6)	83.1 (73.3–90.5)	99.3 (98.8–99.6)	99.2 (98.7–99.5)	98.3 (97.7–98.8)	98.5 (97.9–99.0)
	E	7	2	NA	NA	99.7 (99.3–99.9)	99.9 (99.7–100)	NA	NA
	M	211	186	42.2 (35.4–49.2)	100 (95.9–100)	100 (99.8–100)	95.2 (94.1–96.1)	94.2 (93.1–95.2)	95.4 (94.4–96.2)
	O *	29	23	46.0 (33.4–59.1)	36.5 (24.7–49.6)	100 (99.8–100)	100 (99.8–100)	98.4 (97.7–98.9)	98.1 (97.4–98.6)
	S	47	43	45.0 (35.0–55.3)	90.7 (77.9–97.4)	99.9 (99.6–100)	97.0 (96.2–97.7)	97.3 (96.5–97.9)	96.9 (96.1–97.6)
	A	26	21	64.3 (44.1–81.4)	60.7 (40.6–78.5)	99.6 (99.2–99.8)	99.8 (99.5–99.9)	99.1 (98.6–99.5)	99.3 (98.8–99.6)
	U	27	2	NA	NA	98.7 (98.1–99.2)	99.9 (99.7–100)	NA	NA
Lymphocyte-dominant	N	20	18	85.7 (42.1–99.6)	85.7 (42.1–99.6)	99.4 (99.0–99.7)	99.5 (99.1–99.7)	99.4 (99.0–99.7)	99.5 (99.1–99.7)
	L	1118	1617	63.7 (61.4–65.9)	90.8 (89.3–92.1)	100 (99.5–100)	96.8 (95.2–97.9)	74.1 (72.3–75.8)	92.5 (91.4–93.5)
	E	6	0	0 (0–84.2)	0 (0–84.2)	99.8 (99.5–99.9)	100 (99.9–100)	99.7 (99.4–99.9)	99.9 (99.7–100)
	M	611	516	100 (98.3–100)	100 (98.3–100)	82.4 (80.7–83.9)	86.6 (85.1–88.0)	83.9 (82.4–85.3)	87.8 (86.4–89.0)
	O	245	25	100 (83.9–100)	76.2 (52.8–91.8)	90.8 (89.6–92.0)	99.5 (99.1–99.7)	90.9 (89.7–92.0)	99.4 (98.9–99.6)
	S	80	65	97.4 (86.5–99.9)	53.8 (41.0–66.3)	98.3 (97.7–98.8)	99.8 (99.6–100)	98.3 (97.7–98.7)	98.6 (98.1–99.0)
	A	287	219	100 (76.8–100)	100 (76.8–100)	88.9 (87.6–90.1)	91.7 (90.5–92.7)	89.0 (87.7–90.2)	91.7 (90.5–92.8)
	U	102	9	NA	NA	95.9 (95.0–96.6)	99.6 (99.3–99.8)	NA	NA
Macrophage-dominant	N	49	16	100 (47.8–100)	80.0 (28.4–99.5)	98.0 (97.4–98.6)	99.5 (99.1–99.7)	98.0 (97.4–98.6)	99.4 (99.0–99.7)
	L	112	103	61.7 (54.1–68.9)	58.3 (50.6–65.7)	99.8 (99.5–99.9)	100 (99.7–100)	96.8 (96.0–97.5)	96.7 (95.9–97.4)
	E	1	0	NA	NA	100 (100–100)	100 (99.8–100)	NA	NA
	M	1833	1922	99.3 (98.8–99.6)	100 (99.8–100)	92.6 (89.7–94.9)	74.9 (70.5–78.9)	98.0 (97.3–98.5)	95.2 (94.2–96.0)
	O	5	0	33.3 (4.3–77.7)	0 (0–45.9)	99.9 (99.6–100)	100 (99.8–100)	99.7 (99.4–99.9)	99.7 (99.4–99.9)
	S	61	29	90.7 (79.7–96.9)	46.3 (32.6–60.4)	99.5 (99.0–99.7)	99.8 (99.5–100)	99.2 (98.8–99.6)	98.5 (97.9–99.0)
	A	158	168	46.8 (38.9–54.9)	96.1 (88.9–99.2)	99.9 (99.7–100)	95.6 (94.7–96.4)	96.1 (95.3–96.9)	95.6 (94.7–96.4)
	U	25	6	NA	NA	98.9 (98.4–99.3)	99.7 (99.4–99.9)	NA	NA
Abnormal lymphocyte-dominant	N	61	42	100 (39.8–100)	100 (39.8–100)	99.2 (99.0–99.4)	99.5 (99.3–99.6)	99.2 (99.0–99.4)	99.5 (99.3–99.6)
	L	282	258	100 (96.8–100)	100 (96.8–100)	97.7 (97.3–98.0)	98.0 (97.6–98.3)	97.7 (97.3–98.0)	98.0 (97.7–98.3)
	E	17	0	NA	NA	99.8 (99.6–99.9)	100 (100–100)	NA	NA
	M	924	38	100 (81.5–100)	100 (81.5–100)	87.7 (86.9–88.4)	99.7 (99.6–99.8)	87.7 (87.0–88.5)	99.7 (99.6–99.8)
	O	716	3598	38.4 (36.2–40.6)	100 (99.8–100)	100 (99.9–100)	68.6 (67.4–69.9)	84.4 (83.6–85.3)	76.6 (75.6–77.5)
	S	1084	1992	100 (99.5–100)	100 (99.5–100)	93.9 (93.3–94.5)	80.4 (79.4–81.3)	94.5 (93.9–95.0)	82.2 (81.3–83.1)
	A	2484	1260	100 (95.0–100)	100 (95.0–100)	67.0 (65.9–68.1)	83.8 (82.9–84.6)	67.4 (66.3–68.4)	83.9 (83.1–84.8)
	U	1820	200	100 (95.3–100)	100 (95.3–100)	76.2 (75.2–77.1)	98.3 (98.0–98.6)	76.4 (75.4–77.4)	98.3 (98.0–98.6)
Malignant cell-dominant	N	49	41	79.3 (66.6–88.8)	100 (91.4–100)	99.9 (99.8–100)	99.6 (99.4–99.8)	99.7 (99.4–99.8)	99.6 (99.4–99.8)
	L	329	378	100 (98.3–100)	100 (98.3–100)	97.2 (96.7–97.7)	96.1 (95.5–96.7)	97.4 (96.9–97.8)	96.3 (95.7–96.8)
	E	0	0	NA	NA	100 (99.9–100)	100 (99.9–100)	NA	NA
	M	1210	174	100 (93.8–100)	100 (93.8–100)	73.8 (72.5–75.1)	97.4 (96.8–97.8)	74.2 (72.9–75.5)	97.4 (96.9–97.8)
	O	412	2281	24.7 (22.6–26.8)	100 (99.8–100)	100 (99.9–100)	78.1 (76.5–79.7)	71.8 (70.4–73.1)	86.3 (85.3–87.3)
	S	728	1321	100 (99.3–100)	100 (99.3–100)	95.2 (94.5–95.9)	80.1 (78.8–81.3)	95.8 (95.2–96.4)	82.5 (81.3–83.6)
	A	859	200	100 (96.2–100)	100 (96.2–100)	82.5 (81.3–83.6)	97.6 (97.1–98.0)	82.8 (81.7–83.9)	97.6 (97.1–98.1)
	U	873	65	100 (92.1–100)	95.6 (84.9–99.5)	81.2 (80.1–82.4)	99.5 (99.2–99.7)	81.4 (80.3–82.6)	99.5 (99.2–99.7)

* Other cells include basophils, lymphoma cells, atypical lymphocytes, blasts, and tumor cells. Data are presented as a number or percentage (95% confidence interval). Abbreviations: see Table 1; NA, not available.

**Table 3:** Agreement between DI-60 pre-classification and verification (10 experiment designs for each sample).

Neutrophil-dominant (total cells n = 2100), κ^*^ = 0.81 (0.79–0.84)								
Pre-classification	Verification							
	N (n = 1737)	L (n = 86)	E (n = 2)	M (n = 186)	O (n = 23)	S (n = 43)	A (n = 21)	U (n = 2)
N (n = 1677)	1677	0	0	0	0	0	0	0
L (n = 76)	8	64	0	0	0	4	0	0
E (n = 7)	3	0	2	0	0	1	1	0
M (n = 211)	19	3	0	182	4	1	1	1
O (n = 29)	10	12	0	0	4	3	0	0
S (n = 47)	7	2	0	1	4	28	4	0
A (n = 26)	2	0	0	1	3	4	15	1
U (n = 27)	10	5	0	2	8	2	0	0
Lymphocyte-dominant (total cells n = 2469), κ = 0.58 (0.56–0.61)								
Pre-classification	Verification							
	N (n = 18)	L (n = 1617)	E (n = 0)	M (n = 516)	O (n = 25)	S (n = 65)	A (n = 219)	U (n = 9)
N (n = 20)	12	1	0	4	0	2	0	1
L (n = 1118)	1	1116	0	0	1	0	0	0
E (n = 6)	5	1	0	0	0	0	0	0
M (n = 611)	0	129	0	453	11	1	17	0
O (n = 245)	0	242	0	1	0	0	2	0
S (n = 80)	0	22	0	2	1	44	10	1
A (n = 287)	0	37	0	47	6	13	181	3
U (n = 102)	0	69	0	9	6	5	9	4
Macrophage-dominant (total cells n = 2244), κ = 0.77 (0.73–0.80)								
Pre-classification	Verification							
	N (n = 16)	L (n = 103)	E (n = 0)	M (n = 1922)	O (n = 0)	S (n = 29)	A (n = 168)	U (n = 6)
N (n = 49)	16	0	0	26	0	0	6	1
L (n = 112)	0	88	0	19	0	1	1	3
E (n = 1)	0	0	0	0	0	0	0	1
M (n = 1833)	0	10	0	1816	0	3	4	0
O (n = 5)	0	4	0	0	0	1	0	0
S (n = 61)	0	0	0	31	0	22	8	0
A (n = 158)	0	0	0	11	0	0	147	0
U (n = 25)	0	1	0	19	0	2	2	1
Abnormal lymphocyte-dominant (total cells n = 7388), κ = 0.32 (0.31–0.33)								
Pre-classification	Verification							
	N (n = 42)	L (n = 258)	E (n = 0)	M (n = 38)	O (n = 3598)	S (n = 1992)	A (n = 1260)	U (n = 200)
N (n = 61)	39	0	0	0	0	10	10	2
L (n = 282)	0	225	0	2	11	25	14	5
E (n = 17)	0	0	0	0	11	6	0	0
M (n = 924)	3	5	0	32	615	224	8	37
O (n = 716)	0	6	0	0	646	57	4	3
S (n = 1084)	0	8	0	1	114	941	4	16
A (n = 2484)	0	1	0	0	715	447	1213	108
U (n = 1820)	0	13	0	3	1486	282	7	29
Malignant cell-dominant (total cells n = 4460), κ = 0.29 (0.27–0.30)								
Pre-classification	Verification							
	N (n = 41)	L (n = 378)	E (n = 0)	M (n = 174)	O (n = 2281)	S (n = 1321)	A (n = 200)	U (n = 65)
N (n = 49)	38	0	0	5	0	0	3	3
L (n = 329)	0	294	0	0	3	29	2	1
E (n = 0)	0	0	0	0	0	0	0	0
M (n = 1210)	1	39	0	142	969	57	0	2
O (n = 412)	0	34	0	0	300	76	1	1
S (n = 728)	1	0	0	14	6	696	4	7
A (n = 859)	0	0	0	4	430	218	178	29
U (n = 873)	1	11	0	9	573	245	12	22

κ^*^ is presented with a 95% confidence interval. Abbreviations: see Table 1.

**Table 4:** Comparison of turnaround time between DI-60 and manual counting.

Process Step	TAT (min: s) (Median, IQR)					
	Total (n = 50)	Neutrophil-Dominant (n = 10)	Lymphocyte-Dominant (n = 10)	Macrophage-Dominant (n = 10)	Abnormal Lymphocyte-Dominant (n = 10)	Malignant Cell-Dominant (n = 10)
DI-60						
1. Preparing for scan	0:56 (0:51–1:05)	0:50 (0:49–0:51)	0:49 (0:49–0:51)	1:15 (1:14–1:16)	0:55 (0:55–0:56)	1:04 (1:04–1:05)
2. Scanning ideal zone	0:21 (0:21–0:21)	0:21 (0:21–0:22)	0:21 (0:21–0:21)	0:21 (0:21–0:21)	0:21 (0:21–0:21)	0:21 (0:21–0:21)
3. Pre-classification	2:50 (2:05–6:47)	1:27 (1:20–1:29)	2:10 (2:05–2:13)	2:50 (2:43–3:05)	11:23 (11:08–11:41)	6:36 (6:25–6:47)
4. Verification	2:58 (1:17–4:56)	1:28 (1:11–1:35)	2:58 (2:27–3:03)	0:59 (0:51–1:06)	8:18 (7:44–8:44)	4:31 (4:21–4:56)
Total *	6:28 (5:12–12:53)	4:06 (3:55–4:11)	6:17 (5:43–6:30)	5:27 (5:12–5:50)	21:05 (20:16–21:53)	12:34 (12:29–12:53)
Manual counting						
1. Placing a slide on the microscope	0:05 (0:04–0:05)	0:05 (0:04–0:05)	0:05 (0:04–0:05)	0:05 (0:05–0:06)	0:05 (0:04–0:05)	0:05 (0:05–0:05)
2. Scanning ideal zone	0:07 (0:06–0:08)	0:08 (0:06–0:08)	0:07 (0:07–0:07)	0:07 (0:06–0:08)	0:07 (0:07–0:08)	0:07 (0:07–0:08)
3. Counting cells	1:28 (1:23–1:42)	1:21 (1:19–1:24)	1:24 (1:21–1:25)	1:23 (1:23–1:27)	1:42 (1:39–1:44)	1:59 (1:56–2:09)
4. Recording results	0:11 (0:09–0:13)	0:11 (0:10–0:13)	0:12 (0:10–0:13)	0:11 (0:10–0:12)	0:13 (0:10–0:14)	0:08 (0:08–0:12)
Total *	1:53 (1:46–2:10)	1:45 (1:41–1:49)	1:48 (1:45–1:50)	1:49 (1:46–1:54)	2:06 (2:02–2:11)	2:25 (2:16–2:29)

* Total value means the sum of the four process steps for the DI-60 and manual counting per slide in total and in each group. Abbreviations: TAT, turnaround time; IQR, interquartile range; n, number.

## Data Availability

The data presented in this study are available on request from the corresponding author.
